# Unilateral Myopia With Posterior Staphyloma

**DOI:** 10.7759/cureus.86708

**Published:** 2025-06-25

**Authors:** Malik Muhammad Hamza Khan, Muhammad Rayyan Malik, Muhammad Bilal Malik, Neha Maqsood, Ahmad Burq Maqsood

**Affiliations:** 1 Ophthalmology and Visual Sciences, Aga Khan University Hospital, Karachi, PAK; 2 Medicine, Multan Medical and Dental College, Multan, PAK; 3 Medicine, School of Medicine, University of Bristol, Bristol, GBR

**Keywords:** high myopia, ocular imaging, pathologic myopia, posterior staphyloma, unilateral myopia, unilateral pathological myopia, visual health, visual impairment

## Abstract

Pathological myopia is a major cause of visual impairment and usually affects both eyes. Unilateral presentations are rare, and posterior staphyloma in such cases is even less common. Recognizing these atypical clinical and anatomical features is vital for accurate diagnosis and management.

A woman in her 30s with no significant birth or family history presented with progressive right eye protrusion over one year and gradual, painless vision loss in the same eye over five years. Visual acuity was counting fingers at 4 feet in the right eye and 20/25 in the left. Autorefraction showed -24.75 D spherical and -4.35 D cylindrical in the right eye, consistent with high myopia; the left eye had mild astigmatism. Fundoscopy revealed features of pathological myopia with a posterior staphyloma involving the macula. MRI demonstrated asymmetric right globe enlargement with posterior scleral thinning, while A-scan measured an axial length of 34.00 mm in the right eye versus 24.04 mm in the left. Optical coherence tomography (OCT) and B-scan confirmed retinal and choroidal thinning with scleral curvature. Thyroid orbitopathy and mass lesions were excluded. A diagnosis of unilateral pathological myopia with type 2 posterior staphyloma was made.

This case highlights the uncommon presentation of unilateral pathological myopia with posterior staphyloma, in contrast to the more common bilateral form. Despite extensive evaluation, no clear cause was identified, underscoring the complexity of the condition. Thorough clinical and imaging assessments are essential for accurate diagnosis. Currently, no definitive treatment exists, so management focuses on monitoring and protecting the unaffected eye.

## Introduction

This report describes the process of diagnosis of unilateral pathological myopia with posterior staphyloma. Pathological myopia affects up to 3% of the population [1]. Of these, a very small subset of patients are seen to be affected by unilateral pathological myopia. According to an Egyptian study, only 19 out of 668 (2.8%) patients were found to have unilateral pathological myopia [2]. A further, and even smaller, subset of these patients goes on to develop unilateral staphyloma. We report a case in which, even upon extensive questioning, examinations, and investigations, no causality could be identified to isolate the unilateral cause.

Unilateral pathological myopia is a relatively uncommon condition in contrast to bilateral myopia. The presence of unilateral myopia signifies a difference either in the axial length or the combined refractive power of the cornea and lens between the eyes. According to one study, increased axial length was the major cause of unilateral high myopia (45 out of 48 patients) [3].

A posterior staphyloma is defined as a protrusion of a confined, well-marginated area in the posterior region of the fundus. It is one of the characteristic findings in pathological myopia (unrestrained axial elongation associated with typical findings of the fundus such as posterior staphyloma or myopic maculopathy). It is currently not well understood if pathological myopia and high myopia are due to defects of similar or unrelated genes [4].

## Case presentation

A woman in her 30s, with no significant birth or family history, presented to an ophthalmology clinic with a complaint of gradually increasing protrusion of the right eye for the past year along with painless, gradual vision loss for the last five years. She first noticed decreased vision in her right eye at the age of 10 while in school, and her parents were unsure of any visual issues since birth. There was no associated pain, redness, or itching, and no reported family history of ocular pathology.

Upon examination, there was a positive Naffziger’s sign (noted as forward displacement of the globe). Upon Hertel’s exophalmometry, the right eye corneal apex was noted to be at 17 mm compared to 14mm for the left eye. Vision of the right eye was noted to be counting fingers at four feet compared to 20/25 in the left eye. Extraocular movements were full and normal in both eyes. The conjunctiva and cornea were clear and intraocular pressure (IOP) was within normal limits in both eyes (14 mmHg bilaterally). There was no apparent squint in any of the eyes.

**Figure 1:**
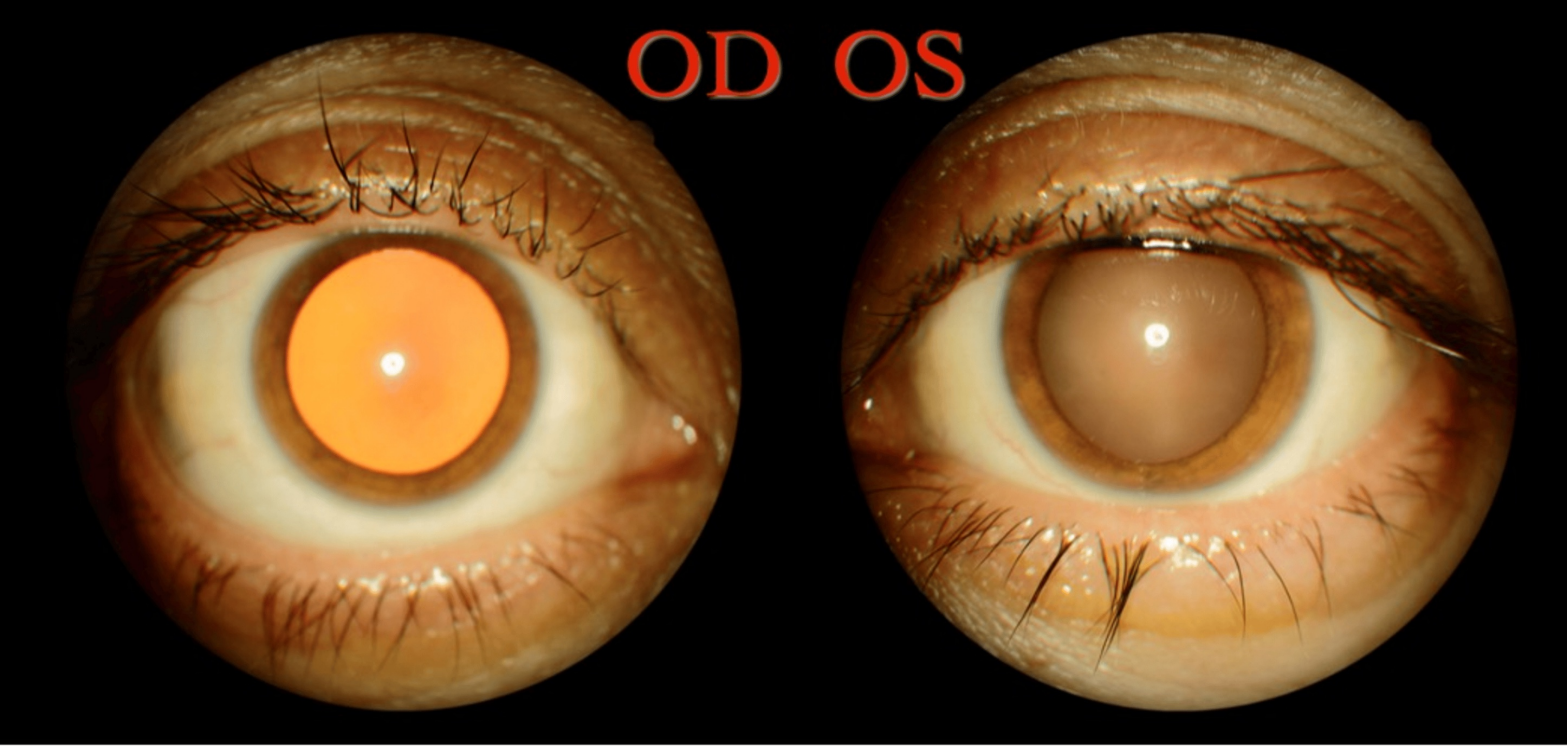
Asymmetric red reflex of highly myopic OD in comparison to normal OS OD: Right eye; OS: Left eye

Red reflex and retinoscopic reflex were indicative of high myopia (Figure 1). Evaluation with an autorefractometer revealed a spherical value of -24.75 and a cylindrical value of -4.35 in the right eye, findings consistent with pathological myopia. The patient’s left eye had a spherical value of -0.25 with a cylindrical value of -2.50 indicating astigmatism. She was using glasses with a plano prescription in the right eye and −0.25/−2.50 × 158 (axis) in the left eye, indicating functional monocular vision from the left eye. The patient demonstrated normal keratometry readings in the right eye (K1: 42.88 D, K2: 44.94 D) and the left eye (K1: 42.50 D, K2: 44.50 D).

**Figure 2:**
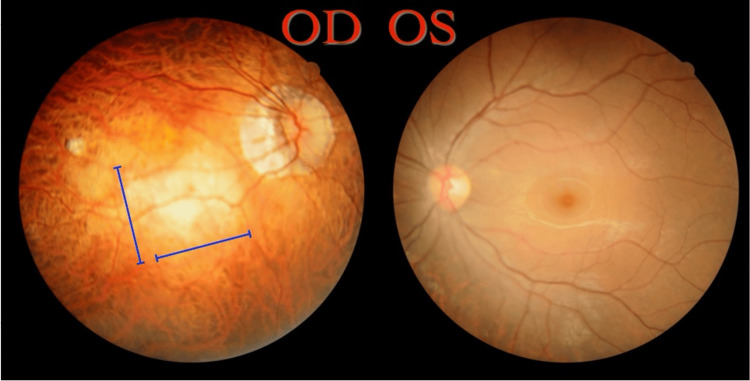
Fundus photographs of both eyes OD: Tilted disc, temporal crescent, tigroid appearance of fundus, central round pale macular lesion with ill-defined borders suggestive of posterior staphyloma (blue lines); OS: Normal OD: Right eye; OS: Left eye

On fundoscopy, using a slit lamp, the right eye showed multiple clinical signs of high myopia. The findings included a tilted disc, a temporal crescent, and thinning of the fundus with tigroid appearance. A round, pale lesion covering the macula from the superior to inferior arcades with blurred borders was noted to be a posterior staphyloma (Figure 2). Laser scars due to prophylactic treatment for retinal tears were found in the periphery. Upon inquiry, the patient reported undergoing two laser treatments within the past year. In comparison, the left eye had a regular appearance on fundoscopy with no evidence of cupping, elevated cup-to-disc ratio, neovascularization, or attenuation. The 360-degree periphery on scleral indentation was normal along with no obvious signs of high myopia pathology. General physical examination was unremarkable.

**Figure 3:**
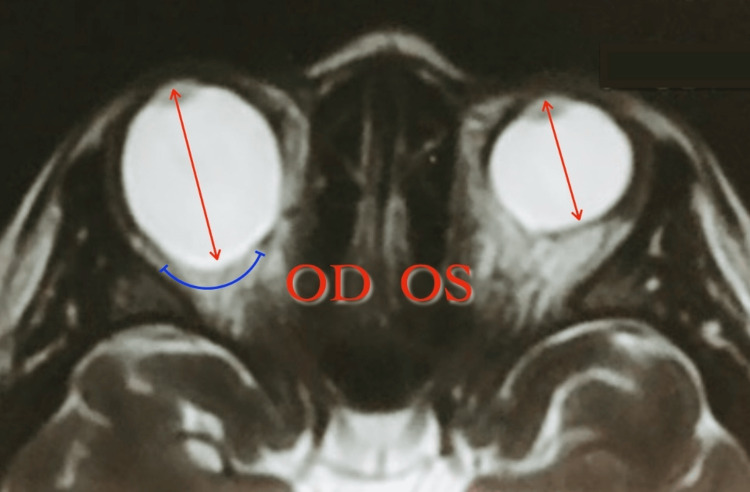
T2-weighted MRI T2-weighted MRI shows a difference in axial length (red arrows) and subtle curvature of posterior staphyloma in the right eye (blue line). Axial length: 34 mm OD; 24.04 mm OS OD: Right eye; OS: Left eye

The patient had no record or history of previous ocular examinations. Upon consultation, serum T3, T4 and thyroid stimulating hormone (TSH) levels were obtained and found to be unremarkable: 1.65 nmol/L, 6.70 ug/dl and 2.945 µIU/ml, respectively. A range of other investigations were also ordered, including MRI, optical coherence tomography (OCT), A-scan, and B-scan. MRI was ordered to rule out thyroid ophthalmopathy and brain masses (Figure 3). The right globe was asymmetrically enlarged, particularly along the anterior-posterior (AP) axis, measuring 3.0 cm in AP length. A focal bulge was identified along the posterior aspect, temporal to the optic disc, accompanied by scleral thinning. The ocular contents of the right eye demonstrated normal signal characteristics. The left globe was normal in size, measuring 2.3 cm in the AP dimension, with ocular content also showing normal signal characteristics. No brain masses were identified. All other findings (including brain findings) were unremarkable and there was no evidence of thyroid ophthalmopathy. The results of the MRI led to the confirmation of the earlier suspected unilateral posterior staphyloma of the right eyeball. 

**Figure 4:**
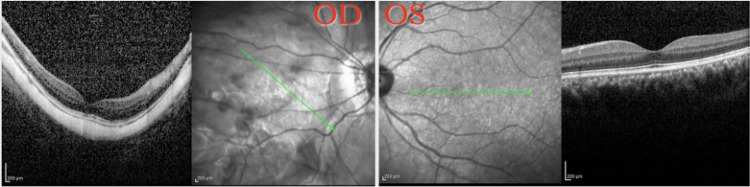
OCT and infrared fundus imaging of both eyes OD: Thinning of the retina and choroid, with a scleral curvature at the location of posterior staphyloma; OS: Normal OCT: Optical coherence tomography; OD: Right eye; OS: Left eye

A significant difference in axial length between the two eyes was noted on A-scan optical biometry, with the right eye measuring 34.00 mm compared to 24.04 mm in the left eye. Anterior chamber depth (ACD) was normal in both eyes - 3.59 mm in the right eye and 3.50 mm in the left. A spectral-domain OCT scan showed thinning of the retina and choroid, with scleral curvature at the location of macular posterior staphyloma. Left eye was normal (retinal layers and foveal contour) and no signs of high myopia were noted (Figure 4). B-scan also confirmed the same contours and findings.

In light of the clinical and imaging findings, a diagnosis of unilateral pathological myopia with posterior staphyloma was made. The differential diagnoses considered, in order of likelihood, included unilateral pathological myopia with posterior staphyloma, thyroid eye disease (ruled out based on unremarkable thyroid function tests), and space occupying intracranial or orbital lesions (excluded through MRI of the brain and orbits). The diagnosis of posterior staphyloma, classified as type 2 (posterior narrow macular staphyloma), was confirmed with clinical examination and OCT. Following the establishment of the diagnosis, multiple counseling sessions were conducted to help the patient understand the nature, course, and management of her condition. Particular care was taken to ensure she understood the unfortunate reality that no definitive treatment was currently available. She was advised to wear protective eyewear and to avoid contact sports, both as part of the management of high myopia and to safeguard her left eye, which remains her only visually functional eye. As there is currently no known treatment for the condition, it was deemed incurable. According to the patient, there has been no progression or complication over the past few years. Nevertheless, she was advised to maintain regular follow-up visits to monitor any potential complications.The patient was not keen to continue with follow up visits due to her geographical location. Other contributory factors included the unavailability of any treatment options, the disease following a non-progressive course and adequate symptomatic management with refractive error correction previously.

## Discussion

In a posterior staphyloma, sudden scleral thinning commences at the edge of the staphyloma due to altered arrangement of collagen fibrils. Choroidal thinning is also noticed most prominently at the staphyloma edge. This is further worsened by additional thinning of the choroid due to increased axial length of the eye. Posterior staphyloma can also be associated with pathologies other than high myopia, e.g., retinitis pigmentosa and defects of the Bruch’s membrane. In these other cases, choroidal thinning is not a prominent finding [5]. Differential diagnoses of a staphyloma could include peripapillary atrophy, tilted-disc syndrome, retinochoroidal coloboma, morning glory syndrome and buphthalmos. These other pathologies have been ruled out in our patient based on OCT interpretation. Evaluating posterior staphylomas using MRI evaluation of posterior staphylomas remains challenging in developing countries due to limited availability and high cost. Latest imaging of posterior staphyloma using widefield OCT has provided tomographic images with a resolution and size that has been unattainable before [6]. The technology is not yet unavailable at our institute. It can also be used to assess damage or changes in the optic nerve as a result of deformities due to pathological myopia. In the future, it may also replace 3D-MRI imaging for assessment and evaluation of posterior staphylomas. The technology is not yet available at our institute.

Upon fundoscopy of eyes with posterior staphyloma, the pathological area is noted to have pallor in association with increased visibility of choroidal vessels. In type 2 posterior staphyloma, areas of ectasia span from the optic disc to the macula with presence of temporal vascular arcades on the upper and lower borders. The shape is shallow or oval with a shallow depth. Margins are noted to be steepest at the disc. The disc usually has an elliptical shape and is tilted temporally along with the presence of a temporal crescent. Retinal vasculature exits the optic disc in a temporal direction; nasal vessels are seen to curve back. Additionally, errors of refraction in posterior staphyloma range from -3.25 to -21.00 diopters with the axial length of eye ranging from 25.7 to 32.0 mm. The presence of a posterior staphyloma is basis enough for a diagnosis of pathological myopia [7].

Eventual macular degeneration in patients with posterior staphyloma can lead to legal blindness. These complications were the reason the patient was asked to adhere to regular follow-up appointments (for lifelong monitoring) along with multiple counselling sessions. Eyes with a posterior staphyloma have been documented to have a higher percentage of equal or more severe diffuse atrophy as compared to eyes without a posterior staphyloma (81.8% vs 54.8%) [8]. In a study conducted on 250 myopic patients with posterior staphylomas in one or both eyes, the incidence of legal blindness was noted to be 19%, with 34.5% of staphylomatous eyes having vision of 20/200 or less [7]. Posterior staphyloma is a feature observed in approximately 55% of eyes with either bilateral or unilateral pathologic myopia [9]. As of yet, no proper clinical or surgical treatment exists for posterior staphylomas. Thus, the course of action mainly relies on careful observation of the patient along with symptomatic treatment. The use of posterior scleral reinforcement, which involves placement of a graft over the posterior pole of the globe to halt the progression of a posterior staphyloma, remains a subject of controversy. Atropine therapy, which has shown efficacy in slowing myopia progression in children but not in adults, was not recommended due to the patient’s age. Understandably, no one therapy has been outlined as the mainstay treatment for posterior staphylomas. This could also be due to the fact that clear focused pathophysiology or causality of pathological myopia and posterior staphylomas is still not clearly understood. Effective interventions could potentially aim towards reducing the incidence or occurrence of complications in pathological myopia (such as posterior staphyloma) or reducing progression of an already present complication.

## Conclusions

This case highlights the occurrence of unilateral pathologic myopia with posterior staphyloma. Despite thorough examinations, no causality was identified, highlighting the enigmatic nature of this condition. Detailed clinical and imaging assessments are crucial for diagnosing such rare presentations and understanding the diverse manifestations of pathological myopia. With no established treatment, management focuses on careful monitoring and symptomatic treatment to mitigate complication risks.
